# SEMI-AUTOMATED 3D SEGMENTATION OF PELVIC REGION BONES IN CT VOLUMES FOR THE ANNOTATION OF MACHINE LEARNING DATASETS

**DOI:** 10.1093/rpd/ncab073

**Published:** 2021-05-25

**Authors:** Julius Jeuthe, José Carlos González Sánchez, Maria Magnusson, Michael Sandborg, Åsa Carlsson Tedgren, Alexandr Malusek

**Affiliations:** Department of Health, Medicine and Caring Sciences, Linköping University, Linköping, Sweden; Department of Health, Medicine and Caring Sciences, Linköping University, Linköping, Sweden; Department of Electrical Engineering, Linköping University, Linköping, Sweden; Department of Health, Medicine and Caring Sciences, Linköping University, Linköping, Sweden; Center for Medical Image Science and Visualization (CMIV), Linköping University, Linköping, Sweden; Department of Health, Medicine and Caring Sciences, Linköping University, Linköping, Sweden; Center for Medical Image Science and Visualization (CMIV), Linköping University, Linköping, Sweden; Department of Health, Medicine and Caring Sciences, Linköping University, Linköping, Sweden; Center for Medical Image Science and Visualization (CMIV), Linköping University, Linköping, Sweden; Department of Medical Radiation Physics and Nuclear Medicine, Karolinska University Hospital, Stockholm, Sweden; Department of Health, Medicine and Caring Sciences, Linköping University, Linköping, Sweden; Center for Medical Image Science and Visualization (CMIV), Linköping University, Linköping, Sweden

## Abstract

Automatic segmentation of bones in computed tomography (CT) images is used for instance in beam hardening correction algorithms where it improves the accuracy of resulting CT numbers. Of special interest are pelvic bones, which—because of their strong attenuation—affect the accuracy of brachytherapy in this region. This work evaluated the performance of the JJ2016 algorithm with the performance of MK2014v2 and JS2018 algorithms; all these algorithms were developed by authors. Visual comparison, and, in the latter case, also Dice similarity coefficients derived from the ground truth were used. It was found that the 3D-based JJ2016 performed better than the 2D-based MK2014v2, mainly because of the more accurate hole filling that benefitted from information in adjacent slices. The neural network-based JS2018 outperformed both traditional algorithms. It was, however, limited to the resolution of 128^3^ owing to the limited amount of memory in the graphical processing unit (GPU).

## INTRODUCTION

Automatic segmentation of human tissues in computed tomography (CT) images finds many applications in medical diagnostic and radiotherapy. Of special interest in this work is the segmentation of bones, which is used for instance in beam hardening correction algorithms in CT or in radiotherapy with protons, where the knowledge of the tissue material allows more accurate estimation of stopping powers. Manual segmentation is very laborious and prone to inter- and intraindividual variability^(1)^. Automatic segmentation addresses these problems, nevertheless a perfect segmentation is difficult to achieve. One reason is the large variety in bones ranging from the dense cortex of cortical bone to the much less dense spongy bone. Additional factors that complicate the task are (1) weak or diffuse boundaries of the bone as a result of the partial volume effect; (2) brightening of tissue between bones close to each other as a result of the low-pass effect of the CT system and (3) holes in the cortical bone for the blood vessels^(2)^.

During the last years, several new automated algorithms for the segmentation of the pelvic and femur bones have been developed. Most of these algorithms either combine several simple methods in order to overcome the drawbacks of the individual algorithms or include prior knowledge by applying a model of the segmented organ. Good performance was also observed for algorithms based on deep learning.

Typical methods that use a model to segment the bones are active shape models^(3)^, statistical shape models^(4)^ and atlas segmentation^(5, 6)^. In active and statistical shape models, the model is created from a training set, which describes the allowed deformations of the model. After the initialization, the model is iteratively adapted to the image content. A large training set allowing an accurate segmentation of the bone is crucial for both methods.

Two algorithms for automated bone segmentation in the pelvic region that do not use a trained model were published by Vasilache and Najarian^(7)^ and Krčah *et al*.^(8)^. The algorithm by Vasilache and Najarian segments the compact bone by a seeded region growing algorithm in 2D. Krčah *et al*. segmented the femur bone by first enhancing the borders of the data by a boundary enhancement filter before the bone was segmented using graph-cut.

In the case of deep learning algorithms, promising results were achieved via convolutional neural networks using two-dimensional kernels in a slice-by-slice segmentation^(9)^ or via a pseudo-3D segmentation^(10)^. The latter approach used the successful 2D U-Net developed by Ronneberger *et al*.^(11)^ for a whole-body segmentation of the skeleton. In the JS2018 algorithm, González Sánchez *et al.*^(12)^ adapted the 3D U-Net developed by Çiçek *et al.*^(13)^ for the segmentation of pelvic bones in dual energy CT.

For bone segmentation in the model-based iterative reconstruction algorithm DIRA^(14)^, the authors developed a 2D hybrid segmentation algorithm MK2014v2^(15)^. DIRA uses material decomposition^(14)^ of the segmented bone into compact bone and bone marrow and thus requires that holes in bones are classified as bone since they may contain bone marrow; this additional requirement complicates direct application of segmentation algorithms designed for other purposes. The MK2014v2 algorithm was later extended to 3D and made more robust. The resulting JJ2016 algorithm^(16)^ was used for the preparation of ground truth for the deep learning-based JS2018 algorithm^(12)^. The aim of this work is to compare the performance of the three algorithms. The main focus is on the JJ2016 algorithm; the description and performance of MK2014v2 and JS2018 algorithms were published elsewhere.

## MATERIALS AND METHODS

### J‌J2016

**Figure 1:**
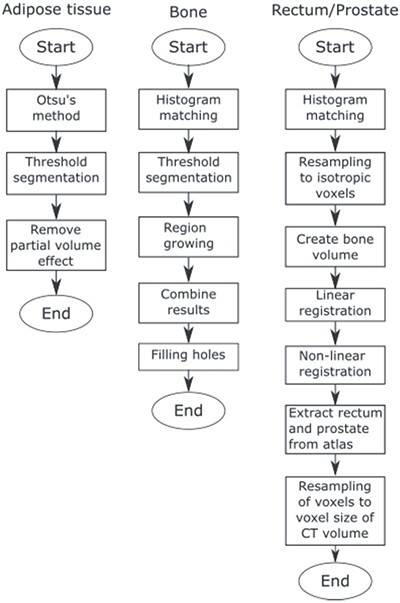
Flowchart of the JJ2016 algorithm.

JJ2016 is a fully automated segmentation algorithm that can segment bones, adipose tissue, rectum and the prostate in single-energy CT images of the pelvic region, see Figure 1. It was developed as a part of the model-based iterative reconstruction algorithm DIRA^(14)^ by extending our previous hybrid algorithm MK2014v2^(17)^ to 3D. In the following text, only the segmentation of bones is described.

The algorithm comprises five steps:

(1) Histogram matching. The intensities of the CT volume are adjusted to match intensities of a reference volume. This reduces the effect of a different tube voltage. Also, the patient table is removed in this step.
(2) Threshold segmentation. The first threshold value selects most of the compact bone. The second threshold value (larger than the first one) selects seed regions for region growing.
(3) Region growing. All adjacent voxels (26 connectivity) with intensity values inside the acceptance range are included.
(4) Results from region growing and threshold segmentation are combined.
(5) Hole filling. Bone parts that are close to each other are merged and the interior of the bone is filled.

The region growing algorithm was inspired by Vasilache and Najarian^(7)^. It uses two criteria, which are calculated in advance to speed up the process in Matlab. The first criterion states that a voxel needs a higher intensity than the mean intensity in its neighbourhood in order to be added to the seed region. It was implemented as follows: (1) A copy of the CT volume is averaged by an *n* × *n* × *n* box filter (*n* = 9 in this study). (2) A mask is created, where voxels that have a higher intensity value in the original CT volume than in the averaged CT volume are set to 1 and the remaining voxels to 0. The resulting local maximum mask contains an accurate binary representation of the compact bone but also many structures from tissues with lower intensity levels. The second criterion states that a voxel needs an intensity above a threshold to be included in the seed region. The region grows when both criteria are met.

### Data processing

Thirty anonymised DECT datasets produced by a Siemens SOMATOM Force scanner at low (mostly 80 kV) and high (mostly 150 kV) tube voltages and corresponding mixed images with the energy weighting factor of mostly 0.3 were obtained from PACS. Voxel sizes ranged from 0.63 × 0.63 × 1.00 mm^3^ to 0.98 × 0.98 × 1.00 mm^3^.

In the first step, the performance of JJ2016 was compared to the performance of the original MK2014v2 by visually assessing the quality of segmentation of eight patient datasets that were used for the development of both algorithms. These datasets were taken at 120 kV using several different scanners; the voxel size ranged from 0.7 × 0.7 × 1.0 mm^3^ to 0.9 × 0.9 × 3 mm^3^. In the second step, the performance of JJ2016 was evaluated via the Dice similarity coefficient on the set of 30 mixed datasets for which the ground truth was obtained by manual segmentation. In the third step, the performance of JJ2016 was compared to the performance of the deep learning-based JS2018 algorithm on 5 of the 30 mixed datasets.

## RESULTS

**Figure 2:**
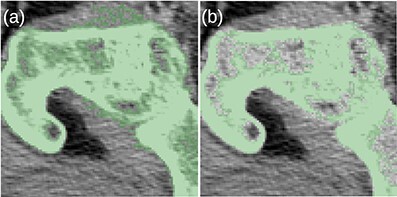
(**a**) The MK2014v2 algorithm misclassified the muscle attached to the bone. (**b**) This problem was mitigated by the JJ2016 algorithm.

**Figure 3:**
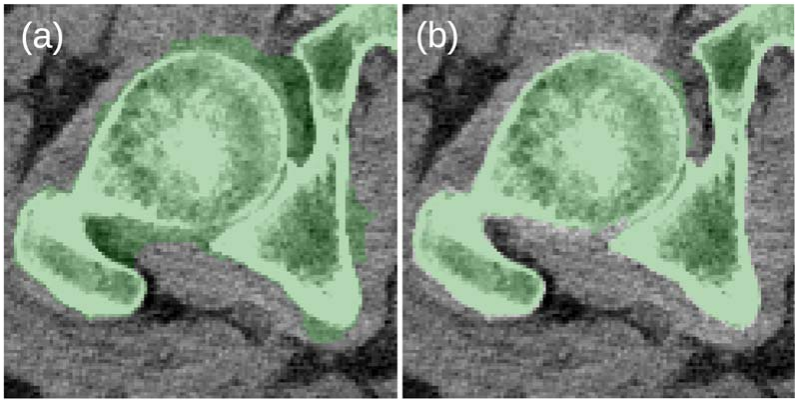
(**a**) Problems with the 2D-based hole filling in MK2014v2. (**b**) The 3D-based hole filling in JJ2016 gave better results.

**Figure 4:**
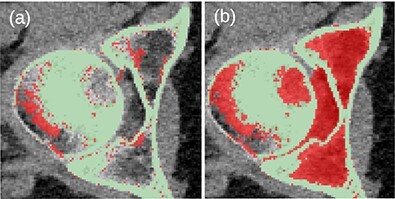
(**a**) Unwanted connection between the head of the femur and the pelvic bone in JJ2016 (red pixels). (**b**) Resulting incorrect hole filling of the region between the two bones (red pixels).

Before the final hole-filling step, the MK2014v2 and JJ2016 algorithms provided similar results; the differences were mostly small. A case with a large difference is shown in Figure 2. Nevertheless, the hole-filling part of JJ2016 was superior to that of MK2014v2, see Figure 3. But even the improved algorithm was not perfect as it sometimes connected the head of the femur with the pelvic bone, see Figure 4a. The space between them was then incorrectly classified as bone marrow.

**Figure 5:**
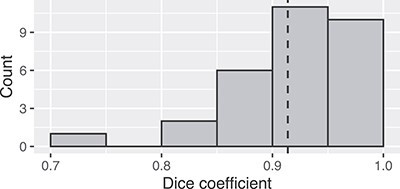
Frequency distribution of Dice coefficients for the 30 patients.

**Figure 6:**
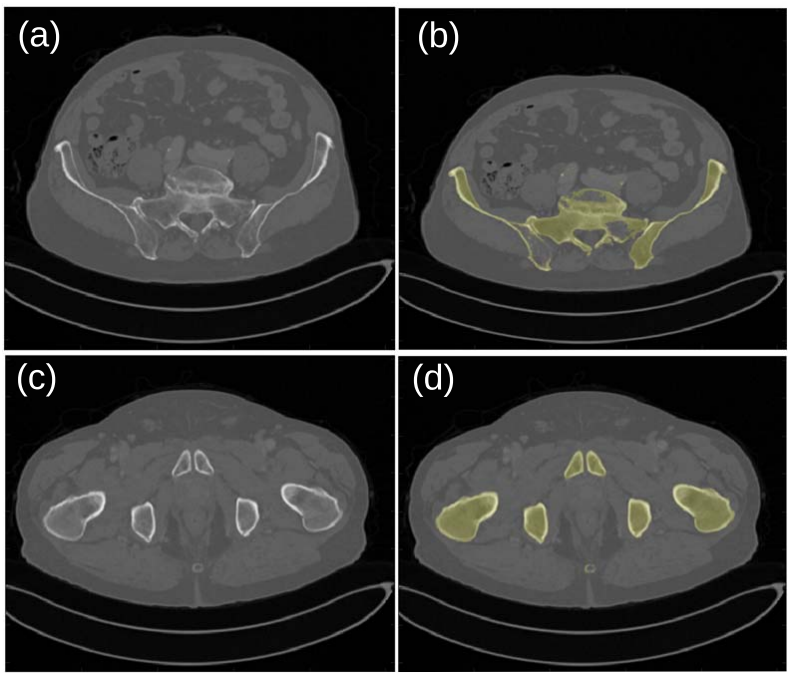
Original images (**a**, **c**) and segmented bone (**b**, **d**) for the patient dataset 26. A hole in the left pelvic bone is not correctly filled in the top-right image.

Figure 5 shows the frequency distribution of Dice coefficients for the 30 segmented patients. The mean and minimum values were 0.914 and 0.714, respectively. Examples of bad (top row) and good (bottom row) segmentation results are shown in Figure 6.

Table 1 shows that the neural network-based JS2018 algorithm provided better results than JJ2016. Its resolution was, however, limited to 128^3^ voxels.

## DISCUSSION

The segmentation of bones is mostly correct when the outer compact bone is thick and dense. Then the inner trabecular bone can be identified by the hole-filling algorithm. When this condition is not met, the hole-filling algorithm may not detect some regions of the bone. Algorithms not using any prior information about the bone have very little chance to succeed in this task. Atlas-based segmentation uses such prior information. Nevertheless, large changes in the bone shape among patients complicate the application of atlases. We have not investigated this approach. Instead, we trained a deep learning neural network. This approach was able to detect the low attenuating bones. Also, it did not classify vessels filled with an iodine contrast agent as bones.

As mentioned in the Methods section, JJ2016 was used for the preparation of the ground truth for the 3D U-Net-based JS2018 algorithm. The data were corrected slice-by-slice using the ITK-SNAP editor. The editing time per dataset varied from 2 h for young and healthy patients to 6 h for patients with osteoporosis, which reduces contrast for the bone. Voxels needing corrections were mainly those in foramina (openings in bones) and regions affected by osteoporosis and contrast agent. The latter was especially problematic for foramen veins. Problems were also encountered in regions with large variability in bone shape and thickness, like sacrum, coccyx and hip joint.

**Table 1:** Dice coefficients for patients 25–30.

Patient	JJ2016	JS2018	Difference
26	0.947	0.968	0.021
27	0.965	0.950	−0.015
28	0.942	0.959	0.017
29	0.965	0.974	0.009
30	0.936	0.976	0.040

Parts of the new hole-filling algorithm in JJ2016 were inspired by the algorithm by Vasilache and Najarian^(7)^, which focuses on identification of broken bones. The additional requirement of hole filling is, however, specific to the JJ2016, see ref.^(16)^ for more information. A comparison with our implementation of the algorithm by Vasilache and Najarian on the set of eight CT datasets showed that JJ2016 performed better when the goal was material decomposition.

## CONCLUSIONS

The JJ2016 segmentation algorithm proved useful for the preparation of a dataset for the training of a 3D U-Net network. Its main advantage compared to the 2D MK2014v2 was a better hole filling. The neural network-based JS2018 provided better segmentation results than both traditional algorithms. It was, however, limited to the resolution of 128^3^. Anonymised original datasets and the manually segmented bones are available from the authors.

## FUNDING

This work was supported by the Vetenskapsrådet (VR-NT 2016-05033).

## CONFLICT OF INTEREST

The authors declare no conflict of interest with regards to this work.
